# Efficacy of interoceptive and embodied rehabilitative training protocol in patients with mild multiple sclerosis: A randomized controlled trial

**DOI:** 10.3389/fneur.2022.1095180

**Published:** 2022-12-21

**Authors:** Teresa Paolucci, Alessandro de Sire, Francesco Agostini, Andrea Bernetti, Angela Salomè, Marta Altieri, Vittorio Di Piero, Antonio Ammendolia, Massimiliano Mangone, Marco Paoloni

**Affiliations:** ^1^Department of Medical, Oral and Biotechnological Sciences (DSMOB), Physical Medicine and Rehabilitation Unit, G. D'Annunzio University, Chieti-Pescara, Chieti, Italy; ^2^Department of Medical and Surgical Sciences, University of Catanzaro “Magna Graecia”, Catanzaro, Italy; ^3^Department of Anatomical and Histological Sciences, Legal Medicine and Orthopedics', Sapienza University, Rome, Italy; ^4^Department of Neurological and Rehabilitation Science, IRCCS San Raffaele, Rome, Italy; ^5^Multiple Sclerosis Center, Sapienza University, Rome, Italy

**Keywords:** balance, exercise, interoceptive awareness, neurocognitive, posture, rehabilitation

## Abstract

**Introduction:**

The aim of this randomized controlled trial was to evaluate the effect of an embodied rehabilitative protocol, in improving interoceptive awareness respect balance and motor performance in patients with mild multiple sclerosis (pwMS).

**Methods:**

In this study patients with relapsing-remitting multiple sclerosis were enrolled. The rehabilitative treatment group (TG) participated in an embodied physiotherapy program consisting of 8 one-hour sessions in groups of 4 patients at a time, 1 per week and 2 one-hour sessions of neuro-cognitive exercise in single session during the rehabilitation program. All pwMS underwent a clinical assessment to measure the interoception sense for the Multidimensional Assessment of Interoceptive Awareness scale, balance for the Tinetti Mobility test and stabilometry, quality of life for the Short Form Health Survey-12 and body image perception for Trunk Appearance Perception Scale and Body Image Scale. All previous scales and tests were performed at baseline (T0), at the end of treatment (T1) and after 2 months of follow up (T2).

**Results:**

Sixty patients were enrolled and randomized into two groups: TG (*n* = 30), aged 43.0 ± 10.2 years, and a control/waiting list (WLG) group (*n* = 30), aged 40.7 ± 10.4 years. Statistically significant improvements in interoceptive awareness, body image perception, balance and quality of life were reported in TG versus WLG (*p* < 0.05).

**Discussion:**

This study suggests that enhancing interoceptive awareness could improve postural balance. Future studies with a larger sample of patients will be needed to better quantify the effects of an embodied rehabilitation.

## 1. Introduction

Multiple sclerosis (MS) is a chronic disease of the central nervous system (CNS) that occurs in people of all ages and races often presenting major limitations of mobility and restriction of participation in activities of daily living (ADL) (1). Therefore, an early detection of functional impairment in patients with MS (pwMS) is crucial to monitor the disease progression and to define an early tailored pharmacological and rehabilitative intervention (2). Rehabilitation, including psychotherapy, symptomatic therapy, and physical activities, is the best form of treatment for the symptoms of MS and for improving motor performances, quality of life, etc. (3, 4). Furthermore, given the dysfunction in the adaptive compensatory mechanisms along the course of disease, rehabilitation is generally more effective in earlier phases of MS (5). Conventional rehabilitation in pwMS patients is based on physical therapies and therapeutic exercise to help the patient develop strategies for dealing with different disabilities interventions aimed at acquiring the maximum possible independence in the Activities of Daily Life (ADL) and return to work (6). The problem of “working with disability” will in pwMS continue to escalate if we do not act (7, 8). In fact, only 37% of those with mild MS are in work. Often, in mild MS, balance, mobility impairments and falls are common problematics, and it can complicate job management and independence (7). Therefore, by proposing early rehabilitation interventions, aimed at improving cognitive and functional skills to strengthen and contain future and more serious balance dysfunctions (reducing the risk of falls and limiting the movement of patients outside the protected environments), it is also possible to effect on autonomy and on returning to work after exacerbations (7, 8). In the early stages of the disease, the dysfunction of postural control, an increased risk of falls, is about 50–80% (1). Body awareness (BA) is an important channel in processing human information for perception and action that interact with balance control: in stroke patients, for example, a good BA was associated with a better postural control (9). MS patients present several alterations of bodily signals (10) and BA (11–15). The exploration of body listening, which we understand as interoceptive awareness, is a little studied topic in MS, especially in the context of rehabilitation. There are various proposals for neurorehabilitation that indirectly have inserted elements of “awareness” of the exercise intended as a neurocognitive task that the patient must solve through the execution of the function, but these aspects have not yet been addressed in patients with MS (6, 16). For example, when MS patients are used somatosensory cues for the perception of body orientation, or motor imagery, the rehabilitator tries to reconstruct a correct movement pattern through a new learning by the patient in pathological conditions, overcoming the discrepancy between perception, action, and posture (6, 17). Also, physiotherapy using MI, with the application of musical and verbal guides, can produce benefits on gait, fatigue and quality of life in pwMS with a low score in the Expanded Disability Status Scale (18). Also, Berthoz (19) remarked, in The Brain's Sense of Movement, the bridge between the interoception of perception and action as the mechanisms that maintain balance and coordinate the movement. On these premises, this study set out to assess the effect of an embodied rehabilitation treatment, that includes training exercises based on the principles of neurocognitive rehabilitation and specifically favoring, as far as possible, the interception and awareness during movement and respecting the principle to learn in pathological conditions (20). The term of embodied was used to define the novelty of the exercise proposal, where the physiotherapist always brought the patient's attention back to listening to the body and awareness of the movement, starting from the important relationship that exists between interoceptive awareness (IA), the sense of body, posture, and action. Therefore, by the present randomized controlled trial (RCT) we aimed to assess the efficacy of an embodied rehabilitation treatment in terms of interoceptive awareness, balance and motor performance in MS patients.

## 2. Materials and methods

### 2.1. Study participants

The protocol of this RCT was created in accordance with the Standard Protocol Items of the Consort Statement (21), registered at clinicaltrials.gov (NCT03711968) and approved by Ethic Committee of the Umberto I University Hospital in Rome (no. 5125).

Patients with the relapsing-remitting form of MS (MS-RR) diagnosed according to McDonald's diagnostic criteria (22) were voluntarily recruited from September to November 2018 and evaluated by a senior neurologist at Umberto I University Hospital in Rome and referred for a physiatrist consultation at the Rehabilitation Outpatient Clinic. Patients were consecutively enrolled and matched pairs randomized into two groups, according to a computer-generated simple randomization list with a 1:1 (block size 4) allocation ratio (software MATLAB R2007b^®^, The Matworks Inc., USA): an embodied rehabilitation treatment group (TG) and a waiting list group (WL = no rehabilitation treatment, patients were free to make exercise at home as global strengthening exercise and walking activity as usual). To respect allocation, black envelopes were used. All participants signed a written informed consent after receiving detailed information about the aim and procedures of the study, which was conducted in accordance with the Declaration of Helsinki (2013). For ethical reasons, to guarantee all patients access to rehabilitation treatment, at the end of the data collection, the WL patients started their rehabilitation program, and their results are independent of this research. Inclusion criteria were: age between 18 and 60 years, clinically definite MS-RR diagnosis, based on the well-established McDonald criteria (23), Expanded Disability Status Scale (EDSS) (24) score of between 0 and 2.5, body mass index (BMI) <30, Mini-Mental State Examination score ≥24 (25). Patients with relapses within the previous 30 days and with a history of psychiatric disorders, such as schizophrenia, bipolar I or II disorder, or substance-abuse disorders, were excluded, as were those with tumors, rheumatological or diabetic conditions, previous surgery on the spine, presence of a pacemaker, cardiovascular disease, or other neurological disorders. Further exclusion criteria were pregnancy and other ongoing rehabilitation treatments. Patients were on a stable FDA-approved disease-modifying therapy regimen for at least 6 months according to the indications of the reference neurologist.

### 2.2. Intervention

**Figure 1:**
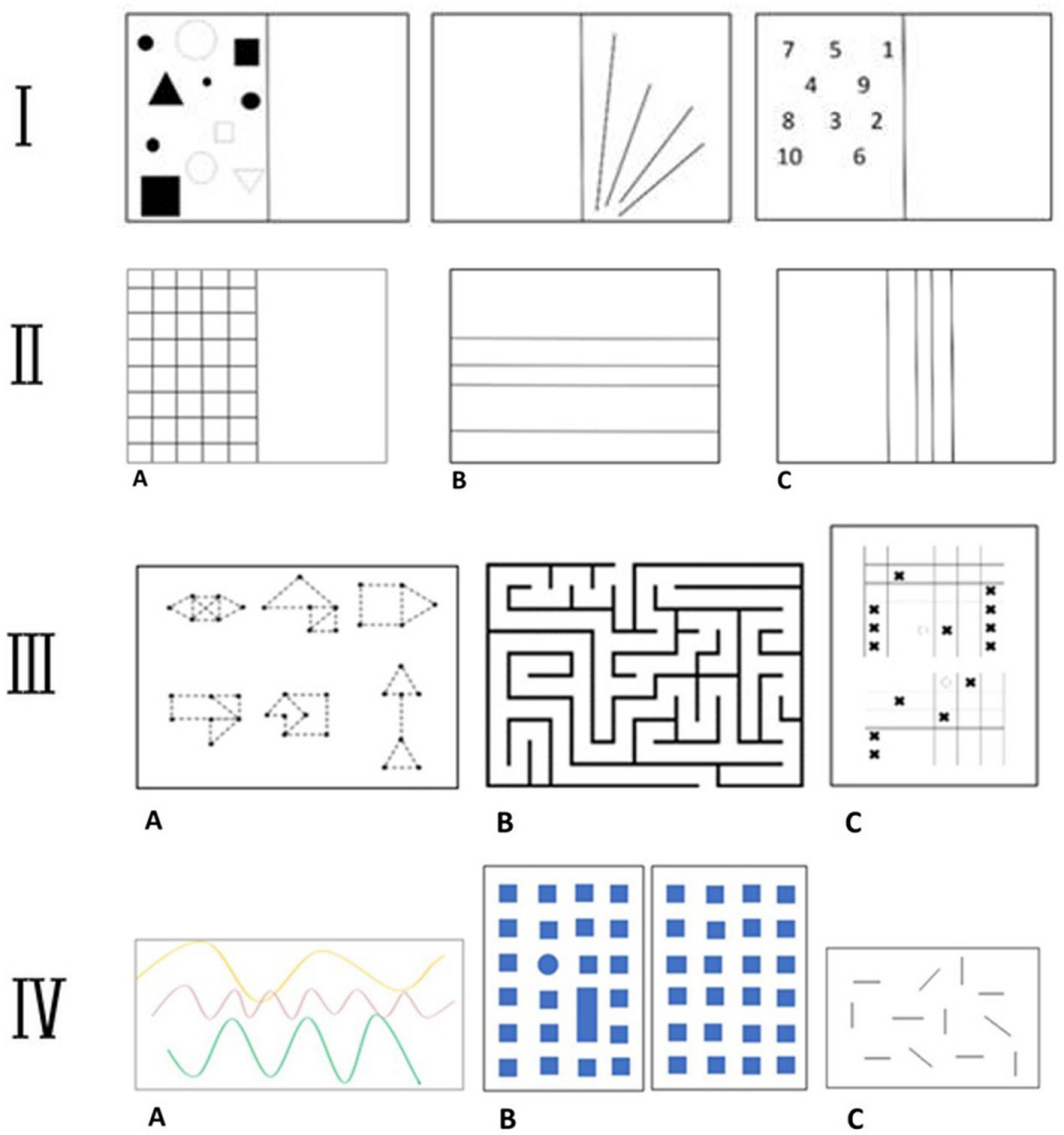
Rehabilitation protocol. **(I)** Cognitive exercise. The physiotherapist gives the patient time to study the image's shapes/lines/numbers (position, size, etc.), before covering up the completed half of the page and asking him/her to draw one or more elements in the same position (short-term memory). **(II)** Cognitive exercise. **(A)** The physiotherapist gives the patient a few minutes to study the image; then, with his/her eyes closed, the patient has to put his/her finger in a requested position (upper limb proprioception). **(B, C)** The patient has to study the distance between the lines. The physiotherapist will then ask him to draw new lines in specific positions (spatial perception). **(III)** Problem-solving test. **(A)** The patient has to join the dots, without going through any of them twice. **(B)** A labyrinth. **(C)** Patient plays against physiotherapist, seeking to the completion of the line with the same symbol. **(IV)** Cognitive exercise. **(A)** The physiotherapist shows the patient three paths painted on the floor. They walk along each of them together paying attention to bends and changes of direction. Then physiotherapist then guides the patient, with eyes closed, along one of the paths and he/she should recognize which it is (motor skills). **(B)** The physiotherapist shows the patient a path on the map. The patient then replicates that path (short-term memory and spatial orientation). **(C)** The Barrage Test (visuo-spatial perception).

The rehabilitation treatment was carried out in groups of five patients, for the first eight sessions, and two single sessions of neurocognitive rehabilitation by two physiotherapists experienced in neurocognitive rehabilitation and by the referring physiatrist (26). The rehabilitation program, aiming to potentiate interoceptive awareness and sensitivity, was based on the following items: (i) posture, (ii) proprioception, (iii) self-body image, (iv) diaphragmatic breathing, (v) relaxation of the body (self-perception), (vi) motor skills, (vii) coordination, (viii) visuo-spatial coordination, and (ix) balance. The program was composed of 8 one-hour sessions of group physical therapy (1 per week) and 2 one-hour sessions of single neurocognitive exercises (1 per month). Each physical therapy session consisted of: postural exercises to increase perception of the body in space, proprioceptive exercises to allow patients to explore and know their own self, visuo-spatial activities to enhance visuo-spatial orientation, coordination and strengthening training to improve balance and strengthen muscles, motor exercises to stimulate memory and coordination, and sporting activities combining fun, motivation and education. To enhance interoceptive awareness (the target of the entire program), the patients initially performed each exercise with EO and then with EC (Table 1). Each exercise session was repeated 8 to 10 times, for two repetitions in the 1st month, and subsequently, for three repetitions in the 2nd month. During the rehabilitation sessions the physiotherapist guided the patient through the voice and the “touch” when and where necessary. At the end of the session performed with the physiotherapist, the patient was instructed to repeat the same exercises at home. At the end of each session, patients performed breathing and relaxation exercises. Neurocognitive exercises were based on the same target following the principles of the Perfetti method (27). The patients worked on proprioception of the upper and lower limbs, short-term memory, spatial perception and orientation and the physiotherapist tested their problem-solving attitude. During the 2 months of rehabilitation treatment the physiotherapist taught the patients how to perform, safely, the same exercises at home for at least two more times a week; moreover, specific cognitive exercises were given designed to train their memory and attention (see Figure 1 for further details). The proposed rehabilitation protocol was designed to help patients improve and focus on interoceptive awareness: posture, body image and body self-awareness, proprioception, visuo-spatial coordination, balance, motor schema, diaphragmatic breathing and relaxation of the body (self-perception). When the exercises were proposed in EC, the patient tried to first imagine the movement, then to make it aware in the body and then, to listen to his/her own body during the execution of the movement itself. Likewise, in the exercises with OE the patient could observe his own movement in the mirror and was always invited to report his own sensations with respect to his/her body in action.

**Table 1:** Rehabilitation plan.

**Rehabilitative sessions**	**Physical therapy program**
First week	- Postural exercises in standing and sitting with eyes open, then with eyes closed. Physiotherapist stimulates a correct stance with the help of the wall, and if necessary, with a light touch Stretching of breathing muscles, in supine position. - Training to stabilize the pelvis and spine in the supine position, in the bridge position, and then a physiological stance with the help of the wall and mirror. - Proprioceptive exercises: in closed eyes condition, the patient has to describe a limb position in space and then actually move the contralateral limb into that same position *(imagine the movement before performing it and describe the sensations and positions assumed)*. (in the supine position, on the side, in the prone position and therefore in an u*p-*right position and while walking). Then, the patient repeats the movement with his/her eyes open.
Second week	- Postural exercises: the same as the previous week, but the physiotherapist starts to use some devices as foam stick as foam stick, soft and tubular balls and soft balls. - Stretching of breathing muscles, in supine position. - Proprioceptive exercises: the physiotherapist moves patient into a position (lying down, sitting or standing). The patient has to maintain it while physiotherapist asks him/her about his/her spatial perceptions. - Looking in the mirror: postural self-correction exercise. - Visuo-spatial exercises: the physiotherapist asks the patient to estimate the distance and the time needed to cover it and then verify both.
Third week	- Strengthening exercises for paravertebral muscles. - Proprioceptive exercises: the patient tries, during movement, to feel the contraction of the muscles that the physiotherapist asks him/her to activate. - Looking in the mirror: the patient has to observe himself/herself and identify his/her incorrect standing position. - Coordination exercises: in standing position the patient throws a ball above his/her head, then claps his/her hands three times before catching it. - Visuo-spatial exercises: with eyes closed, the patient moves around the gym, then the physiotherapist asks him/her where he/she is. - Obstacle course: with eyes open, the patient has to walk on a small tilting platform with the physiotherapist's help, then he/she is required to overcome some easy obstacles.
Fourth week	- Strengthening exercises for the abdominal muscles. - Proprioceptive exercises: patient has to move foam ball around his/her body. - Looking in the mirror: patients have to pass an object (ball/foam stick) to each other, while maintaining eye contact with the mirror. - Obstacle course: with eyes closed, the patient has to walk on a small tilting platform with the physiotherapist's help, and then he/she is required to overcome some easy obstacles. - Coordination exercises: while walking, the patient throws the ball above his/her head, then claps his/her hands three times before catching it. - Motor skills and sporting activity: some exercises from football, volleyball, dance and fencing.
Fifth week	- Proprioceptive exercises: two patients are back-to-back with eyes closed; they have to pass the ball to each laterally, maintaining the position. - Looking in the mirror: the same as the previous week - Coordination exercises: in standing position, the patient throws the ball above his/her head, then turns through 360 degrees before catching it - Motor skills and sporting activity: some exercises from football, volleyball, dance and fencing., dance and fencing.
Sixth week	- Proprioceptive exercises: two patients are back-to-back with eyes closed; they have to pass the ball each other over their head, maintaining the position. - Looking in the mirror: the patient has to stand on one foot, while the physiotherapist draws his/her attention to incorrect muscle contractions. - Coordination exercises: while walking, the patient throws the ball above his/her head, then he turns through 360 degrees before catching it - Obstacle course: with eyes open, the patient has to walk on large tilting platform with the physiotherapist's help, then he/she is required to overcome some easy obstacles and steps. - Motor skills and sporting activity: some exercises from football, volleyball, dance and fencing.
Seventh week	- Proprioceptive exercises: in the fetal position, patient has to roll on the floor. The physiotherapist then helps him/her to straighten his/her position. - Looking in the mirror: patient has to hold a precarious position, while the physiotherapist draws his/her attention to incorrect muscle contractions. - Obstacle course: with eyes closed the patient has to walk on a large tilting platform with the physiotherapist's help, and then he/she is required to overcome some easy obstacles and steps. - Coordination exercises: switching a stick from hand to hand, the patient draws an ideal eight in front of him/her. - Motor skills and sporting activity: some exercises from football, volleyball, dance and fencing.
Eighth week	- Proprioceptive exercises: in a reclining position, the patient has to roll on the floor. The physiotherapist helps him/her to straighten his/her position Coordination exercises: in pairs, patients walk together back-to-back or shoulder to shoulder. They have to do a cross step at the same time; - Obstacle course: the patient has to walk on the large and small tilting platforms with the physiotherapist's help, then he/she is required to overcome some difficult obstacles and high steps. Patient can do this course with eyes closed and/or without the physiotherapist's help. - Motor skills and sporting activity: some exercises from football, volleyball, dance and fencing.

### 2.3. Outcome measures

To minimize bias and maximize the reproducibility of the present RCT, the outcome measures were assessed by physicians unaware of the intervention or control conditions (i.e., by treatment-blinded assessors). Outcome assessments were performed in each group at baseline (T0, before rehabilitative treatment), at the end of 2 months of treatment (T1) consisting of 8 weekly sessions conducted in small groups of five patients, plus two individual sessions (one per month), and after 2 months of follow-up (T2); each treatment session lasted an hour. Primary outcome was the Multidimensional Assessment of Interoceptive Awareness (MAIA) is a scale developed to investigate the different dimensions of IA (28). Among other things, it explores the ability to identify inner sensations and to discern subtle bodily cues indicating varying functional states of the body and the individual's emotional/physiological state. The MAIA scale consists of 32 items divided into eight subscales: Noticing, Not Distracting, Not Worrying, Attention Regulation, Emotional Awareness, Self -Regulation, Body Listening, Trusting. Individual subscale scores range from 5 (a greater degree of awareness) to 0 (a lesser degree of awareness). The maximum total score, indicating the highest degree of self-awareness, is 40 (28).

Secondary clinical outcomes were Tinetti Mobility Test, 12-Item Short Form Health Survey (SF-12), Trunk Appearance Perception Scale (TAPS), and Body Image Scale (BIS). The Tinetti Mobility Test is a recommended instrument for assessing mobility, balance, gait, and fall risk in the elderly. It is composed of a balance subscale (9 items, 16 points) and a gait subscale (8 items, 12 points) (29, 30). The SF-12 (Short Form Health Survey), which derives from the longer the SF-36, measures quality of life through 12 items. Each of its subscales is transformed into a score from 0 to 100, with lower scores indicating increased disability: the Physical and Mental Health Composite Scores (respectively, PCS and MCS) are calculated by specific software. The PCS is calculated by combining the physical functioning, role-physical, bodily pain, and general health scores. The MCS is calculated by a combining the vitality, social functioning, role-emotional, and mental health scores (31). The Trunk Appearance Perception Scale (TAPS) is a validated instrument for testing patient perceived trunk posture. The scale includes three sets of trunk drawings: from the back, from the front and in forward bending position (32). The Body Image Scale (BIS) is a 10-item questionnaire designed to briefly assess body dimension images in cancer patients. It uses a 4-point response scale (ranging from 0 = not at all to 3 = very much) and the final score is the sum of the 10 item scores, and thus ranges from 0 to 30. A score of zero represents no symptoms or distress and higher scores correspond to increasing symptoms and distress or greater body image concerns (33).

### 2.4. Stabilometry evaluation: Balance

A stabilometric platform was used to collect and analyze (Milletrix software ^©^) data on Center of pressure (CoP) oscillation, sway area, length and velocity, both with eyes closed (EC) and with eyes open (EO). The stabilometry test was performed during quiet standing in both conditions (EC and EO) for 51.2 s. After receiving information about the test procedure, the TG and WL patients were instructed to stand erect, but not to attention, with their arms lying along the trunk, their feet at an angle of approximately 30 degrees open toward the front, and their heels aligned in the medio-lateral direction. All tests were performed by the same examiner; thus, the participants were supplied with the same instructions prior to each test. Three tests were conducted for each trial condition (EO and EC), and we report the average scores. In the EO condition, subjects fixated on a mark on a wall 1.5 m away at eye level. The order of trial conditions, EO-EC or EC-EO, was randomized. In order to obtain simulation of self-correction postures, we asked patients to “please stand straight” when performing the test. To minimize external disturbances, the environment was brightly lit naturally and quiet (34). Stabilometry evaluation performed at each time (T0, T1, T2).

### 2.5. Statistical analysis

The sample size was evaluated by considering the item of the MAIA scale: Trusting. We used one-tailed student *t-*tests for dependent samples considering: a power of 95%; α equal to 0.05; we used the following values for item trusting 2.17 ± 1.07 and 3.03 ± 0.97 (35). With these parameters, the required sample is 17 subjects per group. The sample was calculated using the G ^*^ Power Version 3.1.9.2 software. Respect, statistical analyses, the intention-to-treat principle was not considered because the dropout rate was <20% (36). Means and standard deviation values were calculated for all evaluated variables. Related-samples Friedman's two-way analysis of variance by rank test was carried out to assess the scale scores changes in each group over the three timepoints. The Mann-Whitney U test was used to compare scale scores between the two groups at each assessment timepoint. At baseline, we performed unpaired *t-*test to compare the two groups of subjects for age and BMI, we used a Chi-square test (χ^2^) to compare them for gender. All data analysis was performed using IBM SPSS Statistic version 24 software. The *p-*value threshold of significance was set at 0.05 for all tests. The sample size was not calculated, considering this pilot study with respect to the primary outcome considered (MAIA-scale). We also calculated the variations over time (Delta-Δ) for all scales and compared these between the two groups to consider the grou*p-*time interaction of these scales. We did a full statistical analysis for the MAIA scale score. We performed a 2-way mixed ANOVA test with a between subject factor (group) and a within factor (time).

## 3. Results

**Figure 2:**
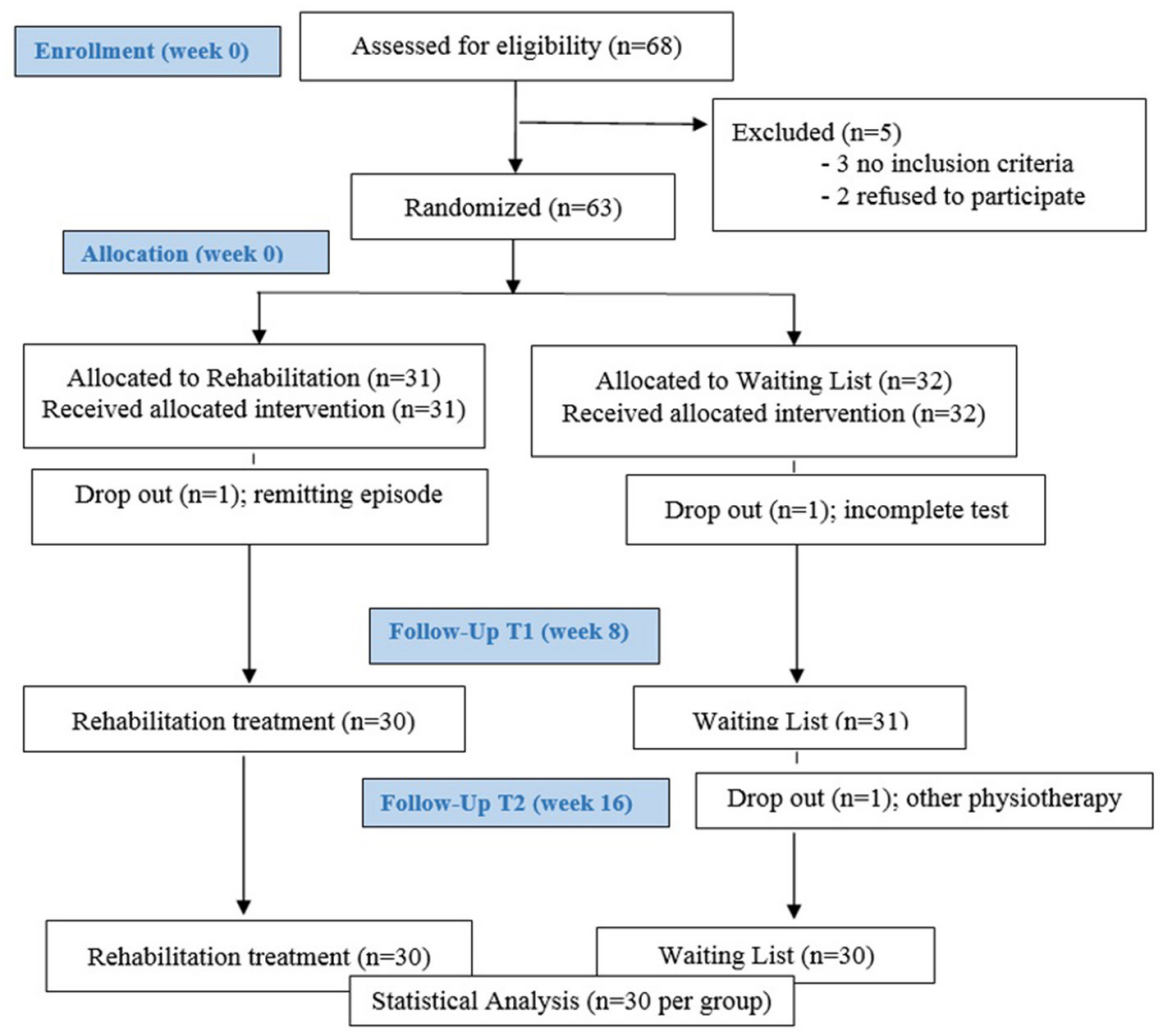
Flow chart.

Sixty-three (*N* = 63) patients were enrolled and randomized into two groups: one submitted to embodied rehabilitation treatment (TG, *N* = 31) and the other comprising patients allocated to the waiting list (WL, *N* = 32). During the two months of rehabilitation treatment, three patients dropped out: two WL patients and one TG patient. Therefore, we analyzed data of 60 patients (42 F, 18 M), 30 undergoing (70% female) the rehabilitation treatment and 30 (70% female) on the waiting list (the study flow diagram is depicted by Figure 2). The two groups were matched for age, gender, BMI and age at diagnosis (years) (see Table 2 for further details). Only one patient had exacerbations of MS symptoms during the rehabilitation intervention. No adverse events occurred during the course of the study.

**Table 2:** Sample characteristics.

**Data**	**Treatment group** ** (*n =* 30)**	**Waiting list group** ** (*n =* 30)**	***p-*value**	**Statistical test**
Gender [male–female (%)]	8–22 (70%)	9–21 (70%)	0.823	χ^2^ test
Age, years (mean ± sd)	43.00 ± 10.16	40.67 ± 10.4	0.383	*t-*test
BMI (kg/m^2^)	24.63 ± 4.3	24.24 ± 3.9	0.383	*t-*test
EDSS [median (first quartiles; third quartiles)]	1.5 (0.5; 2)	1 (1; 1.5)	0.216	*U*-test
Time from diagnosis, years	5.53 ± 3.7	5.20 ± 2.73	0.333	*t-*test
Level of education [graduate–not graduate (%)]	20–10 (33%)	23–7 (23%)		–
Marital status [married–not married (%)]	15–15 (50%)	17−13 (43%)		–
Handedness [right–left (%)]	28–2 (7%)	28–2 (7%)		–
Sporting activity [yes–no (%)]	7–23 (77%)	5–25 (83%)		–
Previous physical therapy [yes–no (%)]	0–30 (100%)	1–29 (97%)		–

M, Male; F, female; C, children; NC, no children; EDSS, Expanded Disability Status Scale score; BMI, body mass index; sd, standard deviation.

### 3.1. Interoceptive awareness

The TG showed statistically significant improvements (*p* = 0.0038) in the MAIA scale total score (MAIA tot): T0 = 21.5 (5.162), T1 = 25.01 (4.902), T2 = 24.05 (4.135) and in five MAIA sub-scales: Noticing (*p* = 0.01), Not Worrying (*p* < 0.001), Emotional Awareness (*p* < 0.001), Self -Regulation (*p* = 0.001) and Body Listening (*p* < 0.001), while in the WL group, there was only a worsening on the Not Distracting item (*p* = 0.002) (Tables 3, 4). Table 5 shows the *post hoc* analysis.

**Table 3:** Mean values, standard deviations and *p-*values of clinical scale scores at three timepoints in the two groups.

**Scale**	**Treatment group**			***p-*value**	**Waiting list group**			***p-*value**
	**T0**	**T1**	**T2**		**T0**	**T1**	**T2**	
**MAIA**							
N	3.03 (0.99)	3.31 (1.02)	3.46 (0.99)	^*^ **0.01**	3.26 (0.91)	3.25 (0.95)	2.87 (1.212)	0.12
ND	2.34 (0.925)	2.26 (1.18)	2.17 (1.21)	0.66	2.218 (0.634)	1.908 (0.0618)	2.009 (0.529)	^*^ ** < 0.01**
NW	2.09 (1.15)	2.67 (0.67)	2.51 (0.95)	^*^ ** < 0.01**	2.40 (0.99)	2.44 (1.01)	2.40 (0.96)	0.79
AR	2.64 (0.77)	2.63 (1.09)	2.69 (0.96)	0.93	2.51 (0.80)	2.31 (0.82)	2.34 (0.92)	0.55
EA	3.16 (1.14)	3.8 (1.11)	3.69 (1.07)	^*^ ** < 0.01**	3.27 (1.05)	3.28 (0.90)	3.14 (0.96)	0.73
SR	2.40 (0.97)	3.28 (1.02)	3.09 (1)	^*^ ** < 0.01**	2.57 (0.85)	2.49 (0.08)	2.48 (0.85)	0.86
BL	2.475 (1.27)	3.25 (1.25)	2.797 (1.13)	^*^ ** < 0.01**	2.48 (0.9)	2.57 (0.97)	2.24 (0.97)	0.06
T	3.34 (1.19)	3.77 (1.08)	3.62 (1.09)	0.33	2.83 (1.14)	2.66 (1.23)	2.67 (1.11)	0.16
Tot.	21.5 (5.16)	25.01 (4.90)	24.05 (4.13)	^*^ ** < 0.01**	21.57 (3.36)	22.54 (6.88)	21.47 (6.23)	0.39
**TINETTI**							
Tot.	24.3 (3.1)	26.7 (2.1)	26.2 (2.4)	^*^ ** < 0.01**	25.1 (3.1)	25.1 (3.1)	24.7 (2.7)	0.17
Balance	13.9 (1.7)	15.4 (1.0)	15.0 (1.2)	^*^ ** < 0.01**	14.3 (1.7)	13.9 (1.9)	13.5 (1.7)	^*^ ** < 0.01**
Gait	10.3 (1.7)	11.4 (1.1)	11.2 (1.2)	^*^ ** < 0.01**	11 (1.3)	11.1 (1.3)	11.2 (1.2)	0.77
TAPS	4.1 (0.7)	4.5 (0.7)	4.3 (0.7)	^*^ ** < 0.01**	4.1 (0.8)	4 (0.8)	4 (0.4)	^*^ ** < 0.01**
BIS	8.4 (7.4)	6.3 (5.7)	5.9 (5.7)	^*^ ** < 0.01**	6.8 (4.1)	7.4 (4.3)	7.7 (4.4)	0.38
SF-12-PCS	36.4 (10.2)	40.8 (10)	40.2 (10.6)	^*^0.01	38.4 (6.6)	38.7 (6.3)	38.4 (6.3)	0.72
SF-12-MCS	41 (10.4)	44.7 (9.4)	43.3 (9.5)	0.15	40.9 (8.2)	40.2 (10.4)	40.7 (9.4)	0.56

MAIA, Multidimensional assessment of interoceptive awareness; N, Noticing; ND, Not distracting; NW, Not worrying; AR, Attention regulation; EA, Emotional awareness; SR, Self-regulation; BL, Body listening; T, Trusting; TAPS, Trunk appearance perception scale; BIS, Body image scale; SF-12, Short-form health survey; PCS, Physical composite score (physical function); MCS, Mental health composite score (mental function).

* Significant.

Bold: Statistically significant.

**Table 4:** The variations over time (Delta-Δ) for all scales.

	**TG median (min; max)**	**WL median (min; max)**	***p*-value**
ΔTinetti T0–T1	3 (0; 5)	0 (−2; 0)	^*^ ** < 0.01**
ΔTinetti T1–T2	0 (−4; 0)	0 (−6; 2)	0.84
ΔTinetti T0–T2	2 (−2; 5)	−1 (−6; 2)	^*^ ** < 0.01**
ΔTAPS T0–T1	0.66 (−1; 1.66)	0 (−3; 1)	^*^ ** < 0.01**
ΔTAPS T1–T2	0 (−1.66; 0.33)	0 (−1; 2.67)	0.92
ΔTAPS T0–T2	0.165 (−1; 1)	−0.33 (−1; 2)	^*^ **0.03**
ΔBIS T0–T1	1.5 (−3; 8)	0 (−7; 5)	^*^ ** < 0.01**
ΔBIS T1–T2	0 (−3;3)	0 (−11; 6)	0.06
ΔBIS T0–T2	2.50 (−4; 11)	−1 (−11; 5)	^*^ ** < 0.01**
ΔMAIA TOT. T0–T1	4.3 (−3.5; 9.14)	0.025 (−4–57; 8.21)	^*^ **0.01**
ΔMAIA TOT. T1–T2	0 (−4.33; 3.05)	−0.13 (−8.15; 5.85)	0.81
ΔMAIA TOT. T0–T2	−3.71 (−7.72; 3.61)	0.46 (−4.75; 5.11)	^*^ ** < 0.01**
ΔSF-12 PCS T0–T1	2.71 (−7.41; 23.38)	0 (−3.56; 3.06)	^*^ ** < 0.01**
ΔSF-12 PCS T1–T2	0 (−17.41; 8.55)	0 (−14.16; 5.08)	0.51
ΔSF-12 PCS T0–T2	2.23 (−14; 16.54)	0 (−14; 7.18)	^*^ **0.02**
ΔSF-12 MCS T0–T1	1.47 (−8.55; 19.76)	0 (−20.75; 16.89)	0.29
ΔSF-12 MCS T1–T2	0 (−23.66; 12.34)	0 (−11.1; 20.48)	0.39
ΔSF-12 MCS T0–T2	1.89 (−27; 19)	4 (−21; 20.48)	0.61

WL, Waiting List Group; TG, Treatment Group; TAPS, Trunk Appearance Perception Scale; BIS, Body Image Scale; MAIA, Multidimensional Assessment of Interoceptive Awareness; SF-12, Short-Form Health Survey; PCS, Physical Composite Score (physical function); MCS, Mental Health Composite Score (mental function);

* Significant.

Bold: Statistically significant.

**Table 5:** Simple main effect analysis for group for MAIA scale.

	**T0**	**T1**	**T2**
TG, mean ± standard deviation	21.50 ± 5.16	25 ± 4.2	24.05 ± 4.13
WL, mean ± standard deviation	21.57 ± 3.36	21.82 ± 4.89	20.65 ± 4.92
*p*-value	0.952	0.014	0.005

TG, Treatment group; WL, Waiting list group; MAIA, Multidimensional assessment of interoceptive awareness.

**Figure 3:**
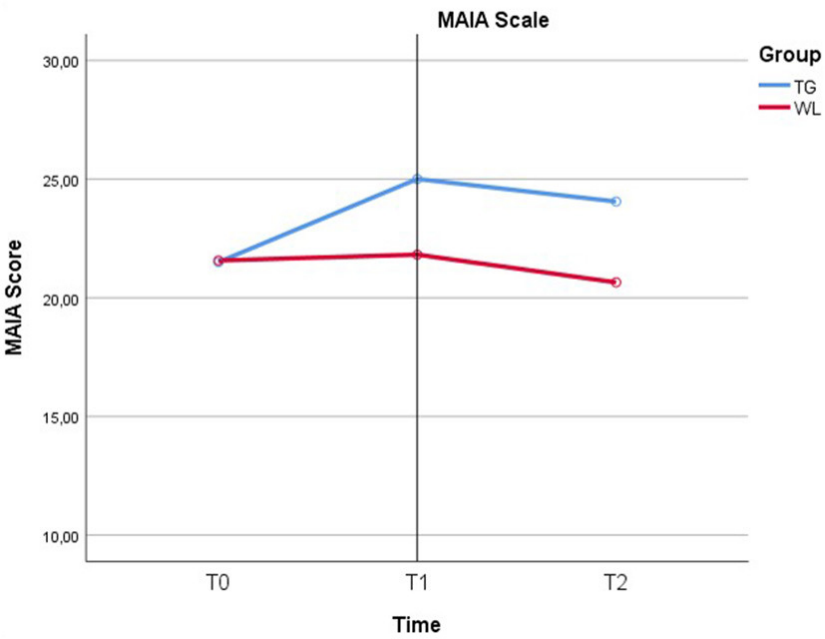
Interaction between the intervention and time on MAIA scale score.

Mauchly's test of sphericity indicated that the assumption of sphericity was violated for the two-way interaction, χ^2^ = 6.37, *p* = 0.041. There was a statistically significant interaction between the intervention and time on MAIA scale score, *F* = 10.02, *p* < 0.001, partial η2 = 0.147 (Figure 3).

We tested for the simple main effects for group that means testing differences in MAIA scale score groups at each category of the within subjects' factor, time (Table 5).

There was a statistically significant effect of time on MAIA scale score in the TG (*p* < 0.01) (Table 6).

**Table 6:** Simple main effect for time for MAIA scale.

**MAIA**	**p total**	**P T0-T1**	**p T1-T2**	**p T0-T2**
	* < 0.01	**0.007**	0.905	**0.045**

MAIA, Multidimensional assessment of interoceptive awareness.

Bold: Statistically significant.

### 3.2. Balance and gait

The Tinetti scale (total score) results showed a statistically significant improvement in the TG patients after rehabilitation treatment and this was preserved at the follow-up, too: T0 = 24.3 (3.17), T1 = 26.7 (2.1), T2 = 26.23 (2.4) (*p* < 0.001). In the WL group, we observed a worsening of Tinetti balance items over the follow-ups: T0 = 14.3 (1.7), T1 = 13.9 (1.9), T2 = 13.5 (1.7) (*p* < 0.001) (Tables 3, 4).

### 3.3. Body image

Statistically significant improvements in the TAPS and BIS scores were observed in the TG: TAPS – T0 = 4.1 (0.7), T1 = 4.5 (0.7), T2 = 4.3 (0.7) (*p* < 0.001); BIS–T0 = 8.4 (7.4), T1 = 6.3 (5.7), T2 = 5.9 (5.7) (*p* < 0.001); instead, the WL group showed a worsening in TAPS scores at T2 and follow-up (Tables 3, 4).

### 3.4. Quality of life

Statistically significant improvements (*p* = 0.0170) in SF-12 physical function scores were recorded at T1 and T2 in the TG: T0 = 36.4 (10.2), T1 = 40.8 (10) and T2 40.2 (10.6) (see Tables 3, 4).

### 3.5. Balance for stabilometry evaluation

In the TG, considering the CoP parameters, we found a reduction of the sway area in the neutral posture with EO after rehabilitation (*p* = 0.01175). In the same group in the neutral posture with EC, we noted a reduction of the sway area (*p* = 0.0396), an increase of the ball length (*p* = 0.00964) and of average velocity (*p* = 0.00025). Furthermore, in these patients we observed an increase in average velocity (*p* = 0.0470) in the self-correction posture with EO, but a decrease of the sway area (*p* = 0.00065) and an increase in average velocity (*p* = 0.01003) in the self-correction posture with EC (Table 7). In the WL, considering the CoP parameters, we observed a statistically significant increase in average velocity (*p* < 0.001) in the self-correction posture with EO (Table 7).

**Table 7:** Mean values, standard deviation and *p-*values of postural parameters measured during the stabilometric platform test at three timepoints in the two groups.

**Stabilometric Evaluation**	**Treatment Group**			***p-*value**	**Waiting List Group**			***p-*value**

	**T0**	**T1**	**T2**		**T0**	**T1**	**T2**	
**Neutral posture EO**							
AS [mm^2^]	371 (873)	64.3 (68.8)	148 (130)	^*^ **0.01**	321 (573)	247 (538)	245 (539)	0.18
BL [mm]	727 (171)	779 (218)	850 (340)	0.68	827 (327)	822 (436)	879 (331)	0.30
CoP aS [mm/s]	14.3 (3.4)	15.2 (4.2)	16 (6.7)	0.29	16.2 (7.6)	16 (6.4)	17.2 (6.5)	0.28
**Neutral posture EC**							
AS [mm^2^]	501 (1268)	273 (418)	83 (163)	^*^0.03	428.5 (725)	322 (684)	230 (453)	0.18
BL [mm]	769 (269)	841 (290)	897 (361)	^*^ ** < 0.01**	835 (364)	844 (345)	879 (366)	0.78
CoP aS [mm/s]	15 (5.2)	16 (5.4)	17.8 (6.8)	^*^ ** < 0.01**	15.2 (7.7)	21 (28)	17.2 (6.5)	0.58
**Self-correction EO**							
AS [mm^2^]	234 (359)	136 (152)	203 (311)	0.32	269 (471)	279 (110)	230 (453)	0.51
BL [mm]	703 (166)	761 (291)	839 (344)	0.12	808 (383)	834 (374)	879 (366)	0.44
CoP aS [mm/s]	13 (3.2)	15.6 (4.7)	16 (6.3)	^*^0.04	15.8 (7.5)	16.4 (7.2)	17 (3.3)	^*^ ** < 0.01**
**Self-correction EC**							
AS [mm^2^]	246 (284)	88.2 (142)	162 (268)	^*^ ** < 0.01**	200 (362)	247 (251)	414 (435)	0.66
BL [mm]	773 (180)	809 (204)	899 (344)	0.07	789 (360)	834 (374)	559 (593)	0.05
CoP aS [mm/s]	15.1 (3.5)	16 (3.6)	17.9 (6.2)	**0.01**	16.8 (8.5)	16.4 (7.2)	18.6 (7.9)	0.14

EO, eyes open; EC, eyes closed; AS, area of sway; BL, ball length; CoP, center of pressure angle; aS, average speed.

* Significant.

Bold: Statistically significant.

### 3.6. Between-group analysis

In the TG we identified statistically significant values with regard to the primary outcome (MAIA scores): total score (T1 *p* = 0.0141; T2 *p* = 0.0345), Emotional Awareness (T1 *p* = 0.0462; T2 *p* = 0.0214), Self-Regulation (T1 *p* = 0.0030; T2 *p* = 0.0168), Body Listening (T1 *p* = 0.0298; T2 *p* = 0.0684).A statistically significant differences in the Tinetti total score (T1 *p* = 0.0072; T2 *p* = 0.0074), Tinetti balance scale (T1 *p* = 0.0007; T2 *p* = 0.0003) and TAPS scores (T1 *p* = 0.0015; T2 *p* = 0.0302) were reported. The BIS scores also differed significantly between the groups at follow-up (T2 *p* = 0.0228). We did not find any statistically significant difference in the SF-12 scores. Regarding postural assessment and stabilometry, we found statistically significant changes in the sway area value in self-correction posture with EO (T1 *p* = 0.0736; T2 *p* = 0.0203) and in path length with EO and EC (T2 *p* = 0.0130).

## 4. Discussion

Our study set out to evaluate the effect of an embodied rehabilitation treatment in mild MS. This treatment was designed to improve interoceptive awareness and enhance balance and motor skills in the early stages of the disease, with a limited and mild disability, in patients who had never yet undergone a rehabilitation process. It is termed embodied because it comprises exercises intended to increase body awareness and, specifically, interoceptive awareness.

In pwMS, the functional reduction represents one of the most disabling aspects since it limits the patient's functionality in ADL, not allowing a complete and timely return to work, fundamental to an individual's social self-determination (6). This loss of autonomy and social self-definition, due to the impossibility in the return to work, lead the patient to completely abandon work activities, as feeling no longer able to do it, and to isolate themselves socially (6). Therefore, by virtue of such a complex pathology and with extremely varied symptoms, all aspects of disability must be investigated and addressed.

In our study, the TG showed improvements on several MAIA subscales, namely Not Worrying, Emotional Awareness, Self-Regulation and Body Listening, both after treatment and at follow-up; these improvements were not observed in the WL group. According to the results, exercises, both with EO and with EC, could be helpful in making patients focus on their own body awareness. The embodied rehabilitation treatment, based on kinesthetic, proprioceptive, and tactile stimuli, could be a new strategy for helping patients to learn about their own bodies. In fact, during each session, the patients focused on their perception of their own body both in neutral posture and during movements, also helped by physiotherapist's voice and touch because some exercises were performed in the EC condition and, in each neurocognitive session, patients performed cognitive training to enhance attention, memory and orientation. Other research studies (37–39) have provided novel evidence about the effectiveness, in the rehabilitation protocol, of training involving specific, graded discrimination tasks with attentive exploration of stimuli with vision occluded, deliberate anticipation and quantitative feedback. Moreover, in pwMS, exercises while walking could improve learning, memory, and hippocampal properties (40) and structured physical activity programs may contribute to cognitive function stability or improvement, while physical activity can enhance balance and gait (41). The results suggested as the embodied rehabilitation program can improve balance, as shown by the Tinetti scale results and interoception as shown by the MAIA scale results. In accordance with Zamariola et al. (42), we observed improved interoception awareness together with TAPS and BIS. Moreover, perception of our own body, feelings, posture, and mental attitude relates to interoception and body image: all of these elements form our own personal identity and contribute to our general well-being (43, 44). Moreover, in self-correction posture with EO, the stabilometric test data showed an increase of CoP velocity and a decrease of sway area, while there was an increase in CoP velocity and a statistically significant reduction in sway area in the same posture with EC. Sway area reduction corresponds to good postural stability maintenance with low energy expenditure, while CoP velocity increase is an effective and quick strategy for constantly seeking center of gravity equilibrium. In neutral posture with EO and with EC, the data showed sway area reduction, but in the EC condition there were also increases in CoP velocity and path length. In summary, better posture control could be achieved through improved interoceptive awareness; sway area reduction was found to be a key result in EC/EO neutral/self-correction postural control in our TG. Postural control relies on the integration of inputs from the visual, somatosensory and vestibular systems, which are frequently impaired in patients with MS and an increase in sway area is an indicator of poor walking and balance capabilities in MS. Therefore, it could be hypothesized that the patient's sense of his own body in space, in static and in dynamic conditions, can represent an additional sense for postural control, particularly considering the virtual reality rehabilitation programs (45, 46). Posturography is considered the gold standard objective measure of standing postural control in pwMS, even in early-stage disease: sway area, length, oscillation, and trajectories are key parameters and appropriate outcomes capable of indicating disability deterioration in MS. Future studies specifically focusing on postural self-correction mechanisms and postural balance would be desirable. This study is not free from limitations. Firstly, the relatively shor*t-*period intervention (i.e., 2 months) and short follow-up observation period, thus not examining the sustainability and durability of the intervention over time. We included patients with an EDSS score between 0 and 2.5 (no or minimal / mild disability in one or two functional systems). Also, there is a lack of comparison with other kinds of exercise or mindfulness treatments. Lastly, patients included in the study did not undergo the same drug therapy. These limitations will be addressed in future studies, with more patients' details, using the same drug therapy, and longer follow-up, to increase the scientific literature on this subject. A desirable goal in future studies, to deepen the results that have been observed, will be to use the ICF model as a reference also as outcomes.

## 5. Conclusions

Taken together, the findings of this double-blind RCT showed that an embodied rehabilitation treatment was effective in terms of enhancing interoceptive awareness and finding a balance between body and mind. Our results are encouraging in favoring modalities of proposals for rehabilitation exercise that focus attention on the awareness of the body in motion, using strategies that make the exercise itself a moment of learning and stimulation, including attention and memory strategies in MS. Also, MS is frequently affecting adults of working age, resulting in a range of physical, cognitive and psychosocial deficits that impact on workforce participation, then it is important to take care of the patient from the early stages of the disease, ensuring a rapid return to work especially after the flare-up phases of the disease. Specific future studies focusing on postural self-correction mechanisms, postural balance in MS patients in order to improve their independence and work capacity despite MS-related disability.

## Data availability statement

The raw data supporting the conclusions of this article will be made available by the authors, without undue reservation.

## Ethics statement

The work has been approved by the Ethical Committee of the Umberto I University Hospital in Rome (no. 5125) and followed ethical guidelines for experimentation with human subjects in accordance with the 1964 Declaration of Helsinki. The patients/participants provided their written informed consent to participate in this study.

## Author contributions

Conceptualization: TP and MP. Methodology: TP and ASi. Investigation: FA, AB, and ASa. Formal analysis: FA, AB, and MM. Data curation: TP, FA, and MM. Writing—original draft preparation: TP, ASi, and FA. Writing—review and editing: AA, MM, and MP. Visualization: AB, ASa, MA, and VD. Supervision: TP, ASi, and MP. All authors read and approved the final version of the manuscript.
